# Breakthrough in large-scale production of iPSCs-derived exosomes to promote clinical applications

**DOI:** 10.3389/fbioe.2023.1257186

**Published:** 2023-08-24

**Authors:** Xiaoxiao Chen, Ke Li, Jiangming Chen, Songwen Tan

**Affiliations:** Xiangya School of Pharmaceutical Sciences, Central South University, Changsha, Hunan, China

**Keywords:** iPSCs, exosomes, 3D culture, biogenesis, large-scale production

## 1 Introduction

Induced pluripotent stem cells (iPSCs) are remarkable scientific advances in both regenerative and personalized medicine. Since they were first reported in 2006, iPSCs have been seem as a promising cell source for drug screening (Rana et al., 2017; Sharma et al., 2017), organoid formation (Nguyen et al., 2021; Wang et al., 2022), autologous cell therapy (Sadeqi Nezhad et al., 2021; Kaneko, 2022; van der Stegen and Rivière, 2023), disease modeling (Rowe and Daley, 2019) and precision medicine. The iPSCs are obtained by transferring and overexpressing four transcription factors—Octamer-binding transcription factor 4 (Oct4), SRY-Box Transcription Factor 2 (Sox2), Kruppel-like factor-4 (Klf4), and cellular Myelocytomatosis oncogene (c-Myc)—into somatic cells (Papapetrou et al., 2009). iPSCs not only provide self-renewing pluripotent stem cells, but also avoids the ethical issues related to embryonic stem cell (ESC) derivation. Thus, iPSCs can be widely in autologous transplantation, significantly reducing the risk of immune response. Meanwhile, iPSCs can be differentiated into various cell types, including erythrocytes (Ebrahimi et al., 2020), hepatocytes (Vallverdú et al., 2021), transplantable hematopoietic stem cells (Rao et al., 2022), insulin-secreting β cells (Maxwell and Millman, 2021), and endothelial cells (Luo et al., 2021), among others.

Exosomes are a type of extracellular vesicles (EVs) released by living cells under normal or pathological conditions, ranging in diameter from 40 to 150 nm, containing secreted active biological molecules. Exosomes play various roles in removing cell waste, regulating cellular communication, delivering biomolecule (e.g., nucleic acid and protein, etc.), and influencing cell state. Researchers have also capitalized on their biocompatibility and low immunogenicity to encapsulate drugs for targeted delivery (Jing et al., 2018; Zhang et al., 2018). Moreover, exosomes exhibit therapeutic properties, including recently discovered anti-oxidative stress effects. For instance, exosomes have been found to inhibit 80% of apoptosis induced by 6-hydroxy-dopamine (6-OHDA) (Jarmalavičiūtė et al., 2015).

Notably, exosomes secreted by iPSCs are applied widely and outstrip the limitation. It provides a viable replacement to cell-free therapy for iPSC medicine. At present, iPSC-Exo shows great potential in skin anti-aging and wound healing. Tang et al. loaded exosomes of mesenchymal stem cell derived from iPSCs (iPSC-MSC) into thermosensitive chitosan-based hydrogel to achieve sustained-release exosomes and effectively downregulate the mRNA expression of collagens (type I α1, type V α1 and type V α2) in corneal stroma, reduce scar formation *in vivo* (Tang et al., 2022). Bo et al. reported that injecting exosomes of iPSCs derived keratinocytes subcutaneously around wound sites could accelerate wound healing (Bo et al., 2022). Besides that, recent studies have evaluated effect of iPSC-Exo on the expression of matrix-degrading enzyme matrix metalloproteinase-1 (MMP-1), matrix metalloproteinase-3 (MMP-3), and Collagen Type I at mRNA Level. After iPSC-Exo treatment, the positive expression of senescence-associated β-galactosidase (SA-β-Gal), a biomarker of senescent human dermal fibroblast (HDF), was significantly reduced (Oh et al., 2018). Meanwhile, establishing iPSC models for complicated diseases is helpful to explore the pathological mechanism and therapeutic regimen. To give an example, bipolar I disorder (BP) is a serious recurrent mood disorder characterized by alternating episodes of mania and depression. Attili et al. transformed the skin of BP patients into iPSCs, and then established an iPSC model of BP using exosomes derived from iPSC astrocytes (Attili et al., 2020).

Although significant progress has been made in clarifying the mechanisms of iPSCs and related exosomes, much of the research remains in the laboratory preparation stage. However, a major obstacle in translating these advances into clinical applications is the inability to consistently and stably obtain high-quality exosomes. The increased production of iPSCs and their exosomes poses numerous challenges. This article will focus on the research progress of optimizing the whole process of upstream and downstream production, and discuss the limitations and challenges faced by iPSCs-derived exosomes in large-scale production and application.

## 2 Biogensis

Biogenesis is the upstream stage of the cell and exosome production process, and the biomass will directly impact the subsequent processes and the final yield. By enhancing the production conditions, we have the opportunity to obtain cells and exosomes with amplified specific properties (Yan and Wu, 2020). Various methods that may facilitate the biogenesis of exosome production are summarized in Table 1.

**TABLE 1:** Various methods that may promote biogenesis of exosome.

Method	Principle	Case	Cell type	Effectiveness	Referrence
3D scaffolds	Increase the culture area	3D-printed scaffold consisting of a pillared array	ECs^a^	EV^b^ production↑	Patel et al. (2019)
Spheroid	Change the cell morphology and density, hypoxia inside the sphere promotes the biogenesis of exosomes	Ultra-low attachment tissue culture flask	HDF^c^	VEGF^d^ and other cytokines↑	Hu et al. (2019)
		Flask coating with poly HEMA^e^	HDF	yield↑ specific property↑	Kim M et al. (2018)
Hydrogel	Simulate the biological niches of stem cells	GelMA^f^	MSCs^g^	improve the cell stemness, yield of 3D-Exo↑	Han et al. (2023)
Microcarriers	Adhesion to microcarriers in suspension cultures promotes the production of exosomes	3D TableTrix™	AD-hMSCs^h^	Operating costs↓	Yan et al. (2020)
Bioreactor Systems	Suspension-Cultivation of iPSCs in bioreactors with controlled conditions	Hollow fiber bioreactor FiberCell System	MSCs	Increase the total yield by 19.4 times	Cao et al. (2020)
		BioBLU 1c disposable bioreactor	iPSCs-MSCs^i^	larger amounts of MSC exosomes were produced over time	Ivirico and Sha (2020)
		The Rotary Cell Culture System	synovial fluid mesenchymal stem cells	Obtained large-scale exosomes	Duan et al. (2022)
Gene modification	Overexpression of exosomal biogenesis-related genes	Overexpression of ALIX^j^	iPSCs^k^	Manipulate iPSCs for production of exosomes	Sundar et al. (2019)
		HSP20^l^-transgenic	Cardiomyocytes	Promotes the production of protective exosomes with TSG101^m^	Zhu et al. (2022)
		Overexpression of TSPAN6^n^	Non-neuronal cells	Size of endosome↑ Number of ILV^o^↑	Blanchette and Rodal (2020)
Stimulation	Mimics the microenvironment of injured tissues, enhancing exosome production	Hypoxia	MSCs	Production efficiency of exosomes↑	Kim M et al. (2018)
		Serum-free culture	iPSCs-MSC	Large-scale production of exosomes	Kim S et al. (2018)
		Acoustic stimulation	mammalian cells	8–10 fold enrichment in the number of exosomes	Ambattu et al. (2020)
		Ion-oxide magnetic nanoparticles	MSCs	Interfering with the endocytosis process and stimulating exosome secretion	Park et al. (2020)
Molecular Interference Chemical Inducers	Regulating mechanisms of exosome secretion, trigger specific signaling pathways and enhancing exosome production	LPS^P^, N-methyldopamine, noradrenaline and adiponectin	MSCs	yield↑ specific property↑	Ti et al. (2015), Wang et al. (2020), Kita and Shimomura (2021)

^a^ ECs: endothelial cells.

^b^ EV: extracellular vesicles.

^c^ HDF: human dermal fibroblast.

^d^ VEGF: vascular endothelial growth factor.

^e^ HEMA: 2-hydroxyethyl methacrylate.

^f^ GelMA: gelatin methacryloyl hydrogel.

^g^ MSCs: mesenchymal stem cells.

^h^ AD-hMSCs: adipose-derived hMSCs.

^i^ iPSCs-MSCs: mesenchymal stem cell derived from induced pluripotent stem cells.

^j^ ALIX: Apoptosis-linked gene-2, interacting protein X.

^k^ iPSCs: induced pluripotent stem cells.

^l^ HSP20: heat shock protein 20.

^m^ TSG101: tumor susceptibility gene 101.

^n^ TSPAN6: Tetraspanin-6.

^o^ ILV: intraluminal vesicles.

^p^ LPS: lipopolysaccharide.

### 2.1 3D culture

Currently, several three-dimensional (3D) culture methods have been introduced, including hanging drop, microwell, hydrogel, scaffold, spinner flask, and hollow fiber bioreactor (Feng et al., 2022). 3D culture enables the harvesting of higher yields and improves the therapeutic effectiveness of exosome products.

Tissue engineering scaffolds made of calcium phosphate, nanofibers and surface-modified polymer nanoparticles can effectively increase the derivatization efficiency. The 3D microenvironment provides support for the assembly of derived organoids (Li Y et al., 2017). In a study by Patel et al., cells were inoculated onto 3D scaffolds, which offered a large cell growth area due to the stacking of pillars. The dynamic culture with active transport of nutrients and gases on the pillars, combined with media perfusion at a flow rate of 4 mL/min in a bioreactor, significantly increased EV production (Patel et al., 2019). Furthermore, microencapsulation provides protection against shear stress and prevents iPSC differentiation. This 3D culture approach holds the potential to achieve large-scale iPSC production (Horiguchi et al., 2014).

Hydrogel can also be used for 3D culture. Studies have shown that the 3D environment constructed by gelatin methacryloyl hydrogel (GelMA) can simulate the biological niches of MSCs, significantly improve the cell stemness of MSCs, and thus increase the yield of 3D-Exo (Han et al., 2023). Additionally, Yu et al. designed a 3D culture model using collagen hydrogel and observed that the 3D microenvironment provided by the hydrogel significantly augmented exosome production and exosomal osteoinductivity both *in vitro* and *in vivo* (Yu et al., 2022).

Suspension culture based on microcarriers is currently the most suitable platform for 3D stem cell culture and has found extensive use in commercial production. Common materials include glass, acrylamide, polystyrene, collagen and alginate (Tavassoli et al., 2018). At the same time, bioreactor matching microcarriers can further increase production. One example of a commercial microcarrier is 3D TableTrix™, which can be used directly without the need for re-sterilization. After adding the medium, it absorbs water and swells, stirring and dispersing into a free single microcarrier. The adipose-derived hMSCs can be amplified more than 500 times in 1 L bioreactor within 11 days (Yan et al., 2020). However, the introduction of microcarriers in the 3D cultivation process may lead to sample contamination with particles of similar size to exosomes, making it difficult to separate them using ultrafiltration or differential centrifugation, resulting in compromised exosome quality. Detailed research into the impact of different materials and microenvironments on exosome quality is necessary to ensure consistent yield and quality (Tavassoli et al., 2018). The significant advantage of spinner flasks and bioreactors is that they are expected to achieve continuous automation and are suitable for industrial mass production of exosomes. The hollow fiber bioreactor FiberCell System (C2025, Frederick) contains thousands of hollow fiber and the hydrophilic polysulfone membrane surface area of up to 3,000 cm^2^ (Cao et al., 2020)**.** Hollow Fiber bioreactor 3D-Exos yields 7.5 times more than 2D-Exos and has greater ability to repair cartilage defects (Yan and Wu, 2020). The ports reserved on the BioBLU 1c disposable bioreactor produced by Eppendorf were used to sample, inoculate and add media, add defoamers and other substances, respectively (Jossen et al., 2019). Researchers at a hospital in Shenzhen have established a detailed protocol for the production of large amounts of Good Manufacture Practice (GMP)-grade exosomes from synovial fluid mesenchymal stem cells using a 3D bioreactor (The Rotary Cell Culture System) (Duan et al., 2022). 3D-exo harvested from Commercial 3D culture platform BioLevitator (Hamilton Bonaduz AG) equipping with alginate micro-carrier cell culture system has better effect of anti-apoptosis (Jarmalavičiūtė et al., 2015).

3D cell spheroids can be harvested using ultra-low attachment tissue culture flasks. Hu et al. collected exosomes derived from these three-dimensional spheres (3D HDF XOs) which expressed significantly higher levels of tissue inhibitor of metalloproteinase-1 (TIMP-1). They demonstrated the efficacy in restoring collagen synthesis ability and anti-aging *in vitro*. The secretion of 3D HDF XOs also changed significantly. For example, the expression of vascular endothelial growth factor (VEGF) in HDFs was 22 times higher in 3D culture system (Hu et al., 2019). A similar spheroid formation was achieved by coating with poly (2-hydroxyethyl methacrylate) (poly HEMA) followed by culture. The expression of F-actin of exosomes was significantly reduced, resulting in the docking of the membrane fusion site and the fusion of the vesicle membrane with the plasma membrane for exocytosis, attributed to the depolymerization of F-actin (Kim M et al., 2018). The change in cell morphology within the 3D cultured spheres, where some cells transitioned from narrow strips to round shapes, led to a decrease in cell culture density and consequently higher exosome production.

### 2.2 Gene modification

Exosomes contain CD63, CD9, CD81, Apoptosis-linked gene-2 interacting protein X (ALIX), heat shock protein 70 (HSP70) and tumor susceptibility gene 101 (TSG101), which can be used as molecular markers for detection (Kalluri and LeBleu, 2020). ALIX overexpression in iPSCs was achieved by CRISPR-Cas9 and lentiviral transduction, resulting in exosomes that were more effective in enhancing cell viability (Sun et al., 2019). Overexpression of Tetraspanin-6 (TSPAN6) in non-neuronal cells increased the size of endosomes and the number of intraluminal vesicles (ILVs), which are related to exosome biogenesis (Blanchette and Rodal, 2020). Exosomes derived from HSP20-transgenic cardiomyocytes protect the heart against stress-induced cardiomyopathy and promote the production of exosomes in combination with TSG101 (Zhu et al., 2022).

### 2.3 Stimulation

Simulating the damaged microenvironment activates stem cells to obtain exosomes with enhanced production and therapeutic functions. Specifically, hypoxia, serum-free cultivation, and other means are conducive to large-scale production (Kim S et al., 2018). Physical stimulation such as the shear stress of bioreactors and acoustic stimulation also activate cells (Feng et al., 2022). In addition, liposome-cell collisions and endocytosis of magnetic nanoparticles combined with magnetic fields promote exosome secretion (Debbi et al., 2022). Certain viewpoints propose that mild hypoxia caused by cell accumulation in the spheroids leads to increased exosome secretion and angiogenesis potential of MSCs (Hazrati et al., 2022). Exosome production is most efficient as the size of the spheroid increases, as reported. This may be due to hypoxia in the center of the sphere, and hypoxic conditions can stimulate stem cells to secrete exosomes. However, the efficiency of exosome production decreases when the sphere size is too large, meaning spheroid cells cannot grow without restriction (Kim M et al., 2018).

### 2.4 Molecular interference

Maintaining the pluripotency of iPSCs is related to the type and concentration of cytokines in the culture medium, such as the transforming growth factor-beta (TGF-β) superfamily proteins, including TGF-β protein, activins, and bone morphogenetic proteins (BMPs), which also play a crucial role in maintaining the pluripotency of iPSCs. However, some studies have pointed out that more than a certain limit of activin A can induce iPSCs differentiation (Horiguchi and Kino-oka, 2021). In addition, the antagonist Noggin, together with bFGF, inhibits the differentiation induction of BMP4 and maintains pluripotency (Xu et al., 2005). Soluble cytokines like lipopolysaccharide (LPS), N-methyldopamine, noradrenaline and adiponectin can affect the size and production of exosomes (Ti et al., 2015; Wang et al., 2020; Kita and Shimomura, 2021). However, the agents may be internalized into exosomes and cause otential physiological hazards (Attili et al., 2020).

## 3 Extraction and purification

After large-scale production of exosomes, they also need to be extracted, purified and properly stored, which will greatly affect the yield of exosomes. Therefore, the optimization of downstream processes is necessary to improve their efficiency (Kimiz-Gebologlu and Oncel, 2022). Currently, the common separation and purification methods include differential ultracentrifugation, ultrafiltration, Size Exclusion Chromatography (SEC), Immunocapture and microfluidic technology (Wang et al., 2021). It is important to note that the extraction process needs to maintain a low temperature to inhibit enzyme activity and prevent the dissociation of exosomes. Differential centrifugation is widely used for general exosome separation. However, it is time-consuming, requires expensive equipment, and has the defect of co-precipitation of non-exosome particles (Li P et al., 2017). Exosome surface proteins specifically bind to cognate antibodies such as CD63, on functionalized magnetic beads to achieve targeted separation. Specific subgroups can be separated, but the sample volume is low. Haraszt et al. extracted exosome CM using tangential flow filtration (TTF) technology, filtered it through a 0.2 mm polyethersulfone (PES) membrane, and passed it through a 500-kDa cutoff TFF cartridge (MidiKros mPES 115 cm^2^, D02-E500-05-S) further increased the production of exosomes produced by 3D cultured cells by 7 times, and the efficacy of siRNA delivery was also increased by seven times (Haraszti et al., 2018). Large-scale collection of natural and synthetic exosomes can be accomplished by microfluidic technology, which can also be applied to hydrogels and bioinks (Amondarain et al., 2023) (Amondarain et al., n. d). An integrated chip combining microfluidics and acoustics can automate rapid exosome separation and can be coupled with downstream exosome quality assessment units, significantly enhancing exosome production efficiency and quality control (Wu et al., 2017). For now, quality control revolves around aspects such as size distribution, protein analysis, and RNA quantification. Ultimately, after undergoing structural and biological stability evaluation, “GMP-grade exosomes” can be obtained (Yao et al., 2019).

## 4 Preservation

Currently, the preservation methods for cells and exosomes mainly include freezing, freeze-drying and spray drying (Li Y et al., 2017). Cryopreservation leads to a substantial decline in the viability and pluripotency of iPSCs cells, along with the loss of exosomes, resulting in reduced yield and limitations in production scale.

Many studies on exploring new cryoprotectants (CPAs) to reduce toxicity and improve the survival rate are carried out (Liu et al., 2021). Disaccharide CPA is the safest solution for exosomes, and trehalose is considered the most effective (Zhang et al., 2020). Recently, trehalose has been proposed as a cryoprotectant, and the morphology and function of EVs after cryopreservation have been greatly improved (Budgude et al., 2021). Mohamed Bahr et al. utilize sodium carboxymethylcellulose (NA-CMC) as a carrier and CPA for exosome-containing medium. This approach demonstrates effective protection even when stored at −20°C, serving as a promising method for the cryopreservation and transportation of exosome products (Bahr et al., 2021). Meanwhile, embedding Dopamine neurons derived from iPSCs into microcapsules can effectively improve the survival rate of cells after cryopreservation, which will be a potential solution for the large-scale cryopreservation of iPSCs (Konagaya and Iwata, 2015).

## 5 Conclusion

With the development of biotechnology, the clinical application of iPSCs and exosomes has gradually dawned. We have described and discussed the critical steps involved in the large-scale production of iPSCs and exosomes, including biogenesis, extraction, purification, and preservation. Additionally, we have summarized the current advances in optimizing these steps. The utilization of 3D culture within a bioreactor tank offers promising opportunities for the mass production of both cell therapy and cell-free therapy based on iPSCs and iPSCs-derived exosomes, but also, with the assistance of gene editing technology, molecule interference, the provision of physical stimulation, and the development of downstream processes that integrate techniques like TTF and microfluidics for separation and purification, along with novel cryopreservation strategies, further expands the prospects for the development of iPSCs-derived exosomes. Nevertheless, there is currently no universally accepted standard specification for the industrialization process of 3D culture. It is anticipated that this approach will be implemented within the biopharmaceutical industry to increase yield and decrease costs. These efforts will contribute to the development of iPSC-related screening models and the market of iPSCs and exosomes.

## References

[B1] Ambattu L. A. Ramesan S. Dekiwadia C. Hanssen E. Li H. Yeo L. Y. (2020). High frequency acoustic cell stimulation promotes exosome generation regulated by a calcium-dependent mechanism. Commun. Biol. 3, 553. 10.1038/s42003-020-01277-6 33020585 PMC7536404

[B2] Amondarain M. Gallego I. Puras G. Saenz-del-Burgo L. Luzzani C. Pedraz J. L. (2023). The role of microfluidics and 3D-bioprinting in the future of exosome therapy. Trends Biotechnol. 2023, 006. 10.1016/j.tibtech.2023.05.006 37302911

[B3] Attili D. Schill D. J. DeLong C. J. Lim K. C. Jiang G. Campbell K. F. (2020). “Astrocyte-derived exosomes in an iPSC model of bipolar disorder,” in Neurodevelopmental disorders: Employing IPSC technologies to define and treat childhood brain diseases. Editors DiCicco-Bloom E. Millonig J. H. (Cham: Springer International Publishing), 219–235. 10.1007/978-3-030-45493-7_8

[B4] Bahr M. M. Amer M. S. Abo-El-Sooud K. Ahmed A. Shehab G. El-Tookhy O. (2021). Proficiency of carboxymethyl cellulose as a cryoprotectant. Clinical and histological evaluation of cryopreserved heterogenous mesenchymal stem cell-exosomal hydrogel on critical size skin wounds in dogs. Int. J. Hematol. Oncol. Stem Cell Res. 15, 178–191. 10.18502/ijhoscr.v15i3.6848 35082999 PMC8748238

[B5] Blanchette C. R. Rodal A. A. (2020). Mechanisms for biogenesis and release of neuronal extracellular vesicles. Curr. Opin. Neurobiol. 63, 104–110. 10.1016/j.conb.2020.03.013 32387925 PMC7483335

[B6] Bo Y. Yang L. Liu B. Tian G. Li C. Zhang L. (2022). Exosomes from human induced pluripotent stem cells-derived keratinocytes accelerate burn wound healing through miR-762 mediated promotion of keratinocytes and endothelial cells migration. J. Nanobiotechnology 20, 291. 10.1186/s12951-022-01504-8 35729564 PMC9210631

[B7] Budgude P. Kale V. Vaidya A. (2021). Cryopreservation of mesenchymal stromal cell-derived extracellular vesicles using trehalose maintains their ability to expand hematopoietic stem cells *in vitro* . Cryobiology 98, 152–163. 10.1016/j.cryobiol.2020.11.009 33253747

[B8] Cao J. Wang B. Tang T. Lv L. Ding Z. Li Z. (2020). Three-dimensional culture of MSCs produces exosomes with improved yield and enhanced therapeutic efficacy for cisplatin-induced acute kidney injury. Stem Cell Res. Ther. 11, 206. 10.1186/s13287-020-01719-2 32460853 PMC7251891

[B9] Debbi L. Guo S. Safina D. Levenberg S. (2022). Boosting extracellular vesicle secretion. Biotechnol. Adv. 59, 107983. 10.1016/j.biotechadv.2022.107983 35588952 PMC9420194

[B10] Duan L. Li X. Xu X. Xu L. Wang D. Ouyang K. (2022). Large-scale preparation of synovial fluid mesenchymal stem cell-derived exosomes by 3D bioreactor culture. J. Vis. Exp. 185, e62221. 10.3791/62221 35969064

[B11] Ebrahimi M. Forouzesh M. Raoufi S. Ramazii M. Ghaedrahmati F. Farzaneh M. (2020). Differentiation of human induced pluripotent stem cells into erythroid cells. Stem Cell Res. Ther. 11, 483. 10.1186/s13287-020-01998-9 33198819 PMC7667818

[B12] Feng Z.-Y. Zhang Q.-Y. Tan J. Xie H.-Q. (2022). Techniques for increasing the yield of stem cell-derived exosomes: what factors may be involved? Sci. China Life Sci. 65, 1325–1341. 10.1007/s11427-021-1997-2 34637101 PMC8506103

[B13] Han M. Zhang Z. Liu Z. Liu Y. Zhao H. Wang B. (2023). Three-dimensional-cultured MSC-derived exosome with hydrogel for cerebral ischemia repair. Biomater. Adv. 149, 213396. 10.1016/j.bioadv.2023.213396 37011424

[B14] Haraszti R. A. Miller R. Stoppato M. Sere Y. Y. Coles A. Didiot M.-C. (2018). Exosomes produced from 3D cultures of MSCs by tangential flow filtration show higher yield and improved activity. Mol. Ther. 26, 2838–2847. 10.1016/j.ymthe.2018.09.015 30341012 PMC6277553

[B15] Hazrati A. Malekpour K. Soudi S. Hashemi S. M. (2022). Mesenchymal stromal/stem cells spheroid culture effect on the therapeutic efficacy of these cells and their exosomes: a new strategy to overcome cell therapy limitations. Biomed. Pharmacother. 152, 113211. 10.1016/j.biopha.2022.113211 35696942

[B16] Horiguchi I. Chowdhury M. M. Sakai Y. Tabata Y. (2014). Proliferation, morphology, and pluripotency of mouse induced pluripotent stem cells in three different types of alginate beads for mass production. Biotechnol. Prog. 30, 896–904. 10.1002/btpr.1891 24585713

[B17] Horiguchi I. Kino-oka M. (2021). Current developments in the stable production of human induced pluripotent stem cells. Engineering 7, 144–152. 10.1016/j.eng.2021.01.001

[B18] Hu S. Li Z. Cores J. Huang K. Su T. Dinh P.-U. (2019). Needle-free injection of exosomes derived from human dermal fibroblast spheroids ameliorates skin photoaging. ACS Nano 13, 11273–11282. 10.1021/acsnano.9b04384 31449388 PMC7032013

[B19] Ivirico J. L. E. Sha M. (2020). Stem cell exosome production on the SciVario® twin, a flexible controller for your bioprocess needs.

[B20] Jarmalavičiūtė A. Tunaitis V. Pivoraitė U. Venalis A. Pivoriūnas A. (2015). Exosomes from dental pulp stem cells rescue human dopaminergic neurons from 6-hydroxy-dopamine–induced apoptosis. Cytotherapy 17, 932–939. 10.1016/j.jcyt.2014.07.013 25981557

[B21] Jing H. He X. Zheng J. (2018). Exosomes and regenerative medicine: state of the art and perspectives. Transl. Res. 196, 1–16. 10.1016/j.trsl.2018.01.005 29432720

[B22] Jossen V. Eibl R. Eibl D. (2019). “Single-use bioreactors – an overview,” in Single‐use technology in biopharmaceutical manufacture (Hoboken, New Jersey: John Wiley & Sons, Inc), 37–52. 10.1002/9781119477891.ch4

[B23] Kalluri R. LeBleu V. S. (2020). The biology, function, and biomedical applications of exosomes. Science 367, eaau6977. 10.1126/science.aau6977 32029601 PMC7717626

[B24] Kaneko S. (2022). Successful organoid-mediated generation of iPSC-derived CAR-T cells. Cell Stem Cell 29, 493–495. 10.1016/j.stem.2022.03.005 35395182

[B25] Kim M. Yun H.-W. Park D. Y. Choi B. H. Min B.-H. (2018). Three-dimensional spheroid culture increases exosome secretion from mesenchymal stem cells. Tissue Eng. Regen. Med. 15, 427–436. 10.1007/s13770-018-0139-5 30603566 PMC6171656

[B26] Kim S. Lee S. K. Kim H. Kim T. M. (2018). Exosomes secreted from induced pluripotent stem cell-derived mesenchymal stem cells accelerate skin cell proliferation. Int. J. Mol. Sci. 19, 3119. 10.3390/ijms19103119 30314356 PMC6213597

[B27] Kimiz-Gebologlu I. Oncel S. S. (2022). Exosomes: large-scale production, isolation, drug loading efficiency, and biodistribution and uptake. J. Control. Release 347, 533–543. 10.1016/j.jconrel.2022.05.027 35597405

[B28] Kita S. Shimomura I. (2021). Stimulation of exosome biogenesis by adiponectin, a circulating factor secreted from adipocytes. J. Biochem. 169, 173–179. 10.1093/jb/mvaa105 32979268

[B29] Konagaya S. Iwata H. (2015). Microencapsulation of dopamine neurons derived from human induced pluripotent stem cells. Biochimica Biophysica Acta (BBA) - General Subj. 1850, 22–32. 10.1016/j.bbagen.2014.09.025 25281770

[B30] Li P. Kaslan M. Lee S. H. Yao J. Gao Z. (2017). Progress in exosome isolation techniques. Theranostics 7, 789–804. 10.7150/thno.18133 28255367 PMC5327650

[B31] Li Y. Li L. Chen Z.-N. Gao G. Yao R. Sun W. (2017). Engineering-derived approaches for iPSC preparation, expansion, differentiation and applications. Biofabrication 9, 032001. 10.1088/1758-5090/aa7e9a 28759433

[B32] Liu X. Hu Y. Pan Y. Fang M. Tong Z. Sun Y. (2021). Exploring the application and mechanism of sodium hyaluronate in cryopreservation of red blood cells. Mater. Today Bio 12, 100156. 10.1016/j.mtbio.2021.100156 PMC8603211 34825160

[B33] Luo J. Shi X. Lin Y. Yuan Y. Kural M. H. Wang J. (2021). Efficient differentiation of human induced pluripotent stem cells into endothelial cells under xenogeneic-free conditions for vascular tissue engineering. Acta Biomater. 119, 184–196. 10.1016/j.actbio.2020.11.007 33166710 PMC8133308

[B34] Maxwell K. G. Millman J. R. (2021). Applications of iPSC-derived beta cells from patients with diabetes. Cell Rep. Med. 2, 100238. 10.1016/j.xcrm.2021.100238 33948571 PMC8080107

[B35] Nguyen R. Da Won Bae S. Qiao L. George J. (2021). Developing liver organoids from induced pluripotent stem cells (iPSCs): an alternative source of organoid generation for liver cancer research. Cancer Lett. 508, 13–17. 10.1016/j.canlet.2021.03.017 33771683

[B36] Oh M. Lee J. Kim Y. J. Rhee W. J. Park J. H. (2018). Exosomes derived from human induced pluripotent stem cells ameliorate the aging of skin fibroblasts. Int. J. Mol. Sci. 19, 1715. 10.3390/ijms19061715 29890746 PMC6032439

[B37] Papapetrou E. P. Tomishima M. J. Chambers S. M. Mica Y. Reed E. Menon J. (2009). Stoichiometric and temporal requirements of Oct4, Sox2, Klf4, and c-Myc expression for efficient human iPSC induction and differentiation. Proc. Natl. Acad. Sci. 106, 12759–12764. 10.1073/pnas.0904825106 19549847 PMC2722286

[B38] Park D. J. Yun W. S. Kim W. C. Park J.-E. Lee S. H. Ha S. (2020). Improvement of stem cell-derived exosome release efficiency by surface-modified nanoparticles. J. Nanobiotechnology 18, 178. 10.1186/s12951-020-00739-7 33287848 PMC7720507

[B39] Patel D. B. Luthers C. R. Lerman M. J. Fisher J. P. Jay S. M. (2019). Enhanced extracellular vesicle production and ethanol-mediated vascularization bioactivity via a 3D-printed scaffold-perfusion bioreactor system. Acta Biomater. 95, 236–244. 10.1016/j.actbio.2018.11.024 30471476 PMC6531369

[B40] Rana P. Luerman G. Hess D. Rubitski E. Adkins K. Somps C. (2017). Utilization of iPSC-derived human neurons for high-throughput drug-induced peripheral neuropathy screening. Toxicol. Vitro 45, 111–118. 10.1016/j.tiv.2017.08.014 28843493

[B41] Rao I. Crisafulli L. Paulis M. Ficara F. (2022). Hematopoietic cells from pluripotent stem cells: hope and promise for the treatment of inherited blood disorders. Cells 11, 557. 10.3390/cells11030557 35159366 PMC8834203

[B42] Rowe R. G. Daley G. Q. (2019). Induced pluripotent stem cells in disease modelling and drug discovery. Nat. Rev. Genet. 20, 377–388. 10.1038/s41576-019-0100-z 30737492 PMC6584039

[B43] Sadeqi Nezhad M. Abdollahpour-Alitappeh M. Rezaei B. Yazdanifar M. Seifalian A. M. (2021). Induced pluripotent stem cells (iPSCs) provide a potentially unlimited T cell source for CAR-T cell development and off-the-shelf products. Pharm. Res. 38, 931–945. 10.1007/s11095-021-03067-z 34114161

[B44] Sharma A. Burridge P. W. McKeithan W. L. Serrano R. Shukla P. Sayed N. (2017). High-throughput screening of tyrosine kinase inhibitor cardiotoxicity with human induced pluripotent stem cells. Sci. Transl. Med. 9, eaaf2584. 10.1126/scitranslmed.aaf2584 28202772 PMC5409837

[B45] Sun R. Liu Y. Lu M. Ding Q. Wang P. Zhang H. (2019). ALIX increases protein content and protective function of iPSC-derived exosomes. J. Mol. Med. 97, 829–844. 10.1007/s00109-019-01767-z 30944935

[B46] Sundar I. K. Li D. Rahman I. (2019). Small RNA-sequence analysis of plasma-derived extracellular vesicle miRNAs in smokers and patients with chronic obstructive pulmonary disease as circulating biomarkers. J. Extracell. Vesicles 8, 1684816. 10.1080/20013078.2019.1684816 31762962 PMC6848892

[B47] Tang Q. Lu B. He J. Chen X. Fu Q. Han H. (2022). Exosomes-loaded thermosensitive hydrogels for corneal epithelium and stroma regeneration. Biomaterials 280, 121320. 10.1016/j.biomaterials.2021.121320 34923312

[B48] Tavassoli H. Alhosseini S. N. Tay A. Chan P. P. Y. Weng Oh S. K. Warkiani M. E. (2018). Large-scale production of stem cells utilizing microcarriers: a biomaterials engineering perspective from academic research to commercialized products. Biomaterials 181, 333–346. 10.1016/j.biomaterials.2018.07.016 30098569

[B49] Ti D. Hao H. Tong C. Liu J. Dong L. Zheng J. (2015). LPS-preconditioned mesenchymal stromal cells modify macrophage polarization for resolution of chronic inflammation via exosome-shuttled let-7b. J. Transl. Med. 13, 308. 10.1186/s12967-015-0642-6 26386558 PMC4575470

[B50] Vallverdú J. Martínez García de la Torre R. A. Mannaerts I. Verhulst S. Smout A. Coll M. (2021). Directed differentiation of human induced pluripotent stem cells to hepatic stellate cells. Nat. Protoc. 16, 2542–2563. 10.1038/s41596-021-00509-1 33864055

[B51] van der Stegen S. J. C. Rivière I. (2023). Unraveling barriers to iPSC-derived CAR-T cell differentiation. Cell Stem Cell 30, 248–249. 10.1016/j.stem.2023.02.004 36868193

[B52] Wang J. Bonacquisti E. E. Brown A. D. Nguyen J. (2020). Boosting the biogenesis and secretion of mesenchymal stem cell-derived exosomes. Cells 9, 660. 10.3390/cells9030660 32182815 PMC7140620

[B53] Wang J. Ma P. Kim D. H. Liu B.-F. Demirci U. (2021). Towards microfluidic-based exosome isolation and detection for tumor therapy. Nano Today 37, 101066. 10.1016/j.nantod.2020.101066 33777166 PMC7990116

[B54] Wang Z. McWilliams-Koeppen H. P. Reza H. Ostberg J. R. Chen W. Wang X. (2022). 3D-organoid culture supports differentiation of human CAR+ iPSCs into highly functional CAR T cells. Cell Stem Cell 29, 515–527.e8. 10.1016/j.stem.2022.02.009 35278370 PMC9119152

[B55] Wu M. Ouyang Y. Wang Z. Zhang R. Huang P.-H. Chen C. (2017). Isolation of exosomes from whole blood by integrating acoustics and microfluidics. Proc. Natl. Acad. Sci. 114, 10584–10589. 10.1073/pnas.1709210114 28923936 PMC5635903

[B56] Xu R.-H. Peck R. M. Li D. S. Feng X. Ludwig T. Thomson J. A. (2005). Basic FGF and suppression of BMP signaling sustain undifferentiated proliferation of human ES cells. Nat. Methods 2, 185–190. 10.1038/nmeth744 15782187

[B57] Yan L. Wu X. (2020). Exosomes produced from 3D cultures of umbilical cord mesenchymal stem cells in a hollow-fiber bioreactor show improved osteochondral regeneration activity. Cell Biol. Toxicol. 36, 165–178. 10.1007/s10565-019-09504-5 31820164 PMC7196084

[B58] Yan X. Zhang K. Yang Y. Deng D. Lyu C. Xu H. (2020). Dispersible and dissolvable porous microcarrier tablets enable efficient large-scale human mesenchymal stem cell expansion. Tissue Eng. Part C. Methods 26, 263–275. 10.1089/ten.tec.2020.0039 32268824

[B59] Yao X. Wei W. Wang X. Chenglin L. Björklund M. Ouyang H. (2019). Stem cell derived exosomes: microRNA therapy for age-related musculoskeletal disorders. Biomaterials 224, 119492. 10.1016/j.biomaterials.2019.119492 31557588

[B60] Yu W. Li S. Guan X. Zhang N. Xie X. Zhang K. (2022). Higher yield and enhanced therapeutic effects of exosomes derived from MSCs in hydrogel-assisted 3D culture system for bone regeneration. Biomater. Adv. 133, 112646. 10.1016/j.msec.2022.112646 35067433

[B61] Zhang M. Jin K. Gao L. Zhang Z. Li F. Zhou F. (2018). Methods and technologies for exosome isolation and characterization. Small Methods 2, 1800021. 10.1002/smtd.201800021

[B62] Zhang Y. Bi J. Huang J. Tang Y. Du S. Li P. (2020). <p&gt;Exosome: a review of its classification, isolation techniques, storage, diagnostic and targeted therapy applications</p&gt;. Int. J. Nanomedicine 15, 6917–6934. 10.2147/IJN.S264498 33061359 PMC7519827

[B63] Zhu M. Wu J. Gao J. (2022). Exosomes for diabetes syndrome: ongoing applications and perspective. Biomater. Sci. 10, 2154–2171. 10.1039/D2BM00161F 35319553

